# Neurovascular unit in diabetic retinopathy: pathophysiological roles and potential therapeutical targets

**DOI:** 10.1186/s40662-021-00239-1

**Published:** 2021-05-01

**Authors:** Shen Nian, Amy C. Y. Lo, Yajing Mi, Kai Ren, Di Yang

**Affiliations:** 1 Department of Pathology, Xi’an Medical University, Xi’an, Shaanxi Province China; 2 Department of Ophthalmology, LKS Faculty of Medicine, The University of Hong Kong, Hong Kong, Hong Kong; 3 Institute of Basic Medicine Science, Xi’an Medical University, Xi’an, Shaanxi Province China; 4 Department of Biochemistry and Molecular Biology, Xi’an Medical University, Xi’an, Shaanxi Province China; 5 Department of Ophthalmology, First Affiliated Hospital of Kunming Medical University, Kunming Medical University, Kunming, Yunnan Province China

**Keywords:** Diabetic retinopathy, Neurovascular unit, Neurodegeneration, Gliosis, Inflammation, treatment

## Abstract

Diabetic retinopathy (DR), one of the common complications of diabetes, is the leading cause of visual loss in working-age individuals in many industrialized countries. It has been traditionally regarded as a purely microvascular disease in the retina. However, an increasing number of studies have shown that DR is a complex neurovascular disorder that affects not only vascular structure but also neural tissue of the retina. Deterioration of neural retina could precede microvascular abnormalities in the DR, leading to microvascular changes. Furthermore, disruption of interactions among neurons, vascular cells, glia and local immune cells, which collectively form the neurovascular unit, is considered to be associated with the progression of DR early on in the disease. Therefore, it makes sense to develop new therapeutic strategies to prevent or reverse retinal neurodegeneration, neuroinflammation and impaired cell-cell interactions of the neurovascular unit in early stage DR. Here, we present current perspectives on the pathophysiology of DR as a neurovascular disease, especially at the early stage. Potential novel treatments for preventing or reversing neurovascular injuries in DR are discussed as well.

## Background

Diabetic retinopathy (DR), one of the common microvascular complications of diabetes, remains the leading cause of visual loss in working-age population (20-74 years) in most industrialized countries [1]. Although some studies indicate that the prevalence of DR in the US has decreased after the improvement in controlling risk factors in diabetes care, the proportion of people with DR is expected to increase globally due to population aging, increasing prevalence of diabetes and longer lifespan of these diabetes patients [1, 2].

Clinically, the diagnosis and classification of DR depend mainly on the retinal microvascular lesions observed by ophthalmic fundus examination [3]. However, numerous investigations suggest that DR is a more sophisticated neurovascular complication, wherein multiple types of cells (neurons, glia, immune and vascular cells) in the retina are disrupted [4]. DR can be generally divided into two stages: the early stage of non-proliferative DR and the advanced stage of proliferative DR. In non-proliferative DR, the classic microvascular features include microaneurysms, hemorrhages, venous beading, hard lipid exudates, cotton-wool spots and intraretinal microvascular abnormalities [1, 3]. In proliferative DR, retinal neovascularization develops owing to ischemia and hypoxia. The new aberrant blood vessels are fragile, leading to vitreous hemorrhage and/or tractional retinal detachment from progressive fibrosis, which may result in severe visual impairment and even blindness [1]. Another important manifestation of DR is diabetic macular edema (DME) present at all stages of DR, although it is more common in severe phases. DME is characterized by the pathological macula thickening due to fluid accumulation in the neural retina resulting from breakdown of blood-retinal barrier (BRB) [5]. DME and retinal neovascularization are the two central reasons for vision loss in patients with DR. Main treatments for DR currently include intravitreal injection of anti-vascular endothelial growth factor (VEGF) and steroid drugs, laser photocoagulation and vitreous surgical interventions, which target only microvascular damages at the later stage when patients have already suffered severe vision impairment [3, 5]. Moreover, many patients fail to respond to intravitreal anti-VEGF therapy, which has been adopted as the current first-line treatment for DR, especially for DME. In these poor responders, the effects of anti-VEGF drugs seem to be transient [6, 7]. Therefore, new therapeutic approaches are highly needed to focus on not only vascular but also nonvascular cells and begin treatment at the early stage. In this review, current views on the pathophysiology of DR as a neurovascular disease at the early stage are discussed. In addition, potential therapeutic strategies arising from laboratory studies and clinical trials are also summarized.

## Main text

A systematic literature search up to September 2020 was performed using the PubMed database for original articles written in English using the following key words: “diabetic retinopathy”, “neurovascular unit”, “neurodegeneration”, “retinal gliosis”, “inflammation”, “neuroprotection” and “treatment”. A further search of references from the retrieved articles was conducted. The relevant articles were selected, studied and summarized.

### The neurovascular unit in retina

The concept of ‘neurovascular unit’, which was first introduced to the central nervous system and then to the retina, refers to the complex functional coupling and interdependency among neuronal, glial, immune and vascular cells that is important to maintain hemostasis and modulate neuronal activities [8–10]. In the retina, all the component cells of the neurovascular unit are integrated to maintain the integrity of the inner BRB and dynamically coordinate local blood flow in response to metabolic demands of the retinal neuropil [11]. Any disruption in the retinal neurovascular unit may have pathophysiological influences on each type of component cells, leading to impairment in the structure and function of microvasculature and neurons. As a complex network, retinal neurons and vasculature are organized in a stratified manner, with superficial vascular plexus lies in the nerve fiber layer (NFL), intermediate vascular plexus reside in the boundary between the inner plexiform layer (IPL) and inner nuclear layer (INL), while deep vascular plexus line the outer surface of the INL [12, 13].

**Fig. 1:**
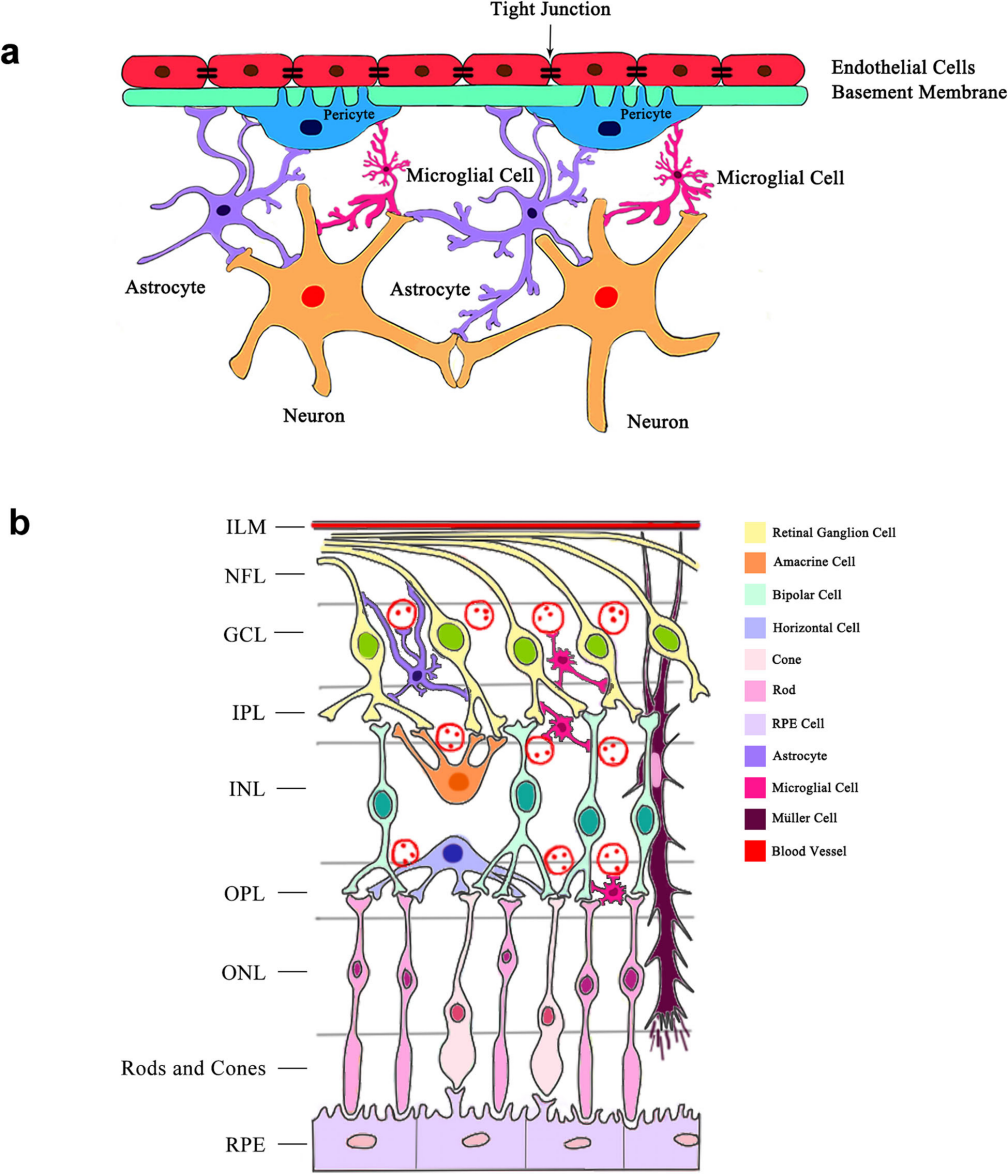
The schematic illustrations of the retinal neurovascular unit and cross-section. (**a**) The schematic illustration of the neurovascular unit in the retina. It is composed of neurons, vascular cells (endothelial cells and pericytes), glia (Müller cells and astrocytes) and related immune cells (microglial cells), forming functional coupling and interactions. Neurons are closely associated with neighboring pericytes, glial cells and microglial cells. Pericytes directly communicate with endothelial cells through peg-socket contacts. Glia and microglial cells are connected with neurons and retinal pericytes. (**b**) The schematic illustration of a retinal cross-section. The cell bodies of retinal neurons are located in the ganglion cell layer (ganglion cells), inner nuclear layer (amacrine cells, bipolar cells, horizontal cells) and outer nuclear cell (rods and cones). Retinal neurons, glial cells, microglial cells and blood vessels are interactively connected. ILM: internal limiting membrane; GCL: ganglion cell layer; OPL: outer plexiform layer; ONL: outer nuclear layer

The retinal neurovascular unit is composed of several types of neurons (ganglion cells, bipolar cells, horizontal cells and amacrine cells), glia (astrocytes and Müller cells), immune cells (microglial cells) and vascular cells (endothelial cells and pericytes) (Fig. 1) [8, 14, 15]. Generally speaking, neurons in the retinal neurovascular unit transmit the electrical impulses converted by photoreceptors from light signals to the brain to form vision. The functions of neurons mainly rely on the blood vessels to supply nutrients and oxygen while eliminating metabolic wastes and carbon dioxide [13]. Furthermore, neurons can regulate local blood flow through glial cells and pericytes to maintain their functions [16].

Müller cells are the major support glial cells in the retina [17]. Their processes wrap around the blood vessels and coordinate vascular responses to provide nutrients to the outer retina, maintain extracellular pH value, recycle transmitter and transport metabolites [17]. Astrocytes encircle the neuronal axons and vessels to form an irregular network to support the integrity of the inner BRB [18]. They may function partially the same as Müller cells and play a role in the maintenance of vascular tone by autoregulation [19].

Microglial cells are in direct contact with neurons and retinal pericytes [15]. As highly specialized phagocytic cells, microglia continuously monitor local synaptic activity and sweep away dying cells as well as metabolic debris [20, 21]. In physiological conditions and acute inflammation, microglial cells produce anti-inflammatory cytokines and neurotrophic factors that support neural retina health [22]. However, in a chronic retinal disease such as DR, they will secrete a large number of pro-inflammatory cytokines, affecting the hemostasis of neurovascular unit [23, 24].

Vascular endothelial cells form a monolayer that covers the inner surface of the vascular lumen. The endothelium is a semi-selective barrier, known as the inner BRB, regulating the flux of fluid and macromolecules between the blood and neural retina by the intercellular tight junctions [25]. Pericytes, which envelope retinal capillaries, are intimately associated with neighboring endothelial cells, neurons, glial cells and microglial cells [14]. Thus, pericytes are crucial in maintaining the neurovascular unit and inner BRB [26]. Despite being separated from endothelial cells by the basement membrane, pericytes are directly connected with endothelial cells via peg-socket contacts at discrete points in the basement membrane [26–28]. In addition to structural support for blood vessels, these contractile pericytes also control blood flow in response to intricate systemic and local messenger molecules [29]. Extracellular matrix (ECM) plays a role in the neurovascular unit too, of which various isoforms such as fibronectin, vitronectin, laminin and collagen IV constitute the basement membrane [30]. ECM is critical to maintain proper neurovascular unit functions since it provides a beneficial environment for the interaction of endothelial cells and pericytes, diffusion of ions, neurotransmitters and ATP [31]. The integrated activity of neurons, glia, microglial, vascular cells and ECM is crucial for the normal function of the retina; however, much work needs to be done to further elucidate the relationships between these components in the neurovascular unit, for instance, the mechanisms of astrocytes interfere with neurons and other astrocytes, the activity of the ECM within the context of the neurovascular unit.

### Pathology related to neurovascular unit in DR

With the improvements of retinal imaging techniques and deep investigations into the early cellular changes, it has been demonstrated that the dysfunction of retinal neurovascular unit plays an important role in the development and progression of DR [13–16, 32, 33]. Additionally, other cell types that are not components of the neurovascular unit such as immune cells and RPE cells are also involved in DR [15, 34, 35] (Table 1).

**Table 1:** Pathological features of the neurovascular unit in diabetic retinopathy (DR)

Pathological Changes	Characteristics	References
Neurodegeneration	Loss of retinal ganglion cells and amacrine cells	[36–40]
	Decrease of NFL, IPL and INL	[37, 38, 40–42]
Reactive gliosis	Activation of astrocytes and Müller cells	[15, 43, 44]
	Death of Müller cells	[45]
Microvascular Pathology	Impaired neurovascular coupling	[46, 47]
	Basement membrane thickening	[48–50]
	Loss of pericytes	[16, 51, 52]
	Formation of microaneurysms	[53, 54]
	Reduction of tight junctions between endothelial cells and apoptosis of endothelial cells	[52, 55–57]
	Breakdown of inner BRB	[55–58]
Immuno-inflammation	Leukostasis	[59–61]
	Activation of microglial cells	[62–64]
	Production of inflammatory cytokines (TNFα, IL-1β, IL-6, IL-8, MCP-1, VEGF)	[65–69]
RPE and Choroid Pathology	Damage of transportation of ions and water in RPE cells	[70, 71]
	Decrease of 11-cis retinal produced in RPE cells	[71, 72]
	Upregulation of cytokines secreted by RPE cells (VEGF, PDGF, TNFα, IL-6, IL-8,)	[73]
	Reduction of tight junctions between RPE cells	[34, 74–76]
	Breakdown of outer BRB	[34, 74–76]
	Choroidal degeneration (decreased choroidal thickness, increased Bruch’s membrane thickness, aneurysms, choroidal neovascularization)	[77–79]

*NFL*= nerve fiber layer; *IPL*= inner plexiform layer; *INL*= inner nuclear layer; *BRB*= blood-retinal barrier; *TNFα*= tumor necrosis factor alpha; *IL-1β*= interleukin-1beta; *IL-6*= interleukin-6; *IL-8*= interleukin-8; *MCP-1*= monocyte chemoattractant protein 1; *VEGF*= vascular endothelial growth factor; *RPE*= retinal pigment epithelium; *PDGF*= platelet-derived growth factor

#### Neurodegeneration in DR

Since Wolter reported the death of retinal ganglion cells and degeneration of the INL in postmortem eyes of people with diabetes in 1961, there is accumulating evidence that neurodegeneration also occurs in diabetic retina, probably even prior to visible microvascular pathologies in DR [36–38]. Increased number of apoptotic neurons has been widely accepted as the most important feature of neurodegenerative disease. In diabetic murine models, apoptosis of retinal neurons, mainly the retinal ganglion and amacrine cells, have been detected before overt microvascular lesions [39]. Another study conducted by Bogdanov et al. also demonstrated apoptosis of retinal ganglion cells in db/db mice, an animal model of spontaneous type 2 diabetes [40]. Similar results were observed as well in several studies examining the retinas of individuals with diabetes [37, 80]; altered expression of some proteins was reported to be linked with retinal neurodegeneration in diabetics, such as somatostatin (SST), apolipoprotein A1, fibrinogen A, pigment epithelial derived factor (PEDF), interphotoreceptor retinol binding protein (IRBP) [81, 82]. The progressive loss of retinal ganglion cells lead to a significant reduction of neuroretinal thickness in both diabetic patients and experimental animal models of diabetes [40, 41, 83]. Furthermore, although patients with advanced DR exhibited increased thickness of whole retina due to inner retinal hemorrhage or retinal edema, decrease in combined thickness of NFL and IPL as well as INL thickness was revealed in type I diabetic patients without or with minimal DR using optical coherence tomography imaging [42].

The structural alterations mentioned above cause retinal dysfunctions, which have been well documented in patients with no or minimal microvascular retinopathy, in some cases even existing before the onset of diabetes [84, 85]. Impairment of retinal functions corresponds to a broad range of manifestations, including reduction of mean amplitudes in pattern electroretinogram (ERG), delayed scotopic implicit time in multifocal ERG, decreased oscillatory potential and b-wave amplitudes, abnormal dark adaption and color vision, loss of contrast sensitivity [84, 86–91]. Diabetes-induced retinal neurodegeneration may be further associated with the development of microvascular abnormalities in DR. This notion is supported by clinical studies showing that the retinal region where neurodegeneration was detected by multifocal ERG would develop microvascular lesions after 12 months [88, 92]. Retinal neurons, such as photoreceptors, may act as the crucial source of oxidative stress and local inflammation [93]. In addition, semaphorin 3A secreted by neural retina may contribute to the breakdown of the BRB via conjugation with its receptor neuropilin-1 [94]. The pathogenic contributions of neurons in DR and the underlying mechanisms of neurodegeneration, including oxidative stress, glutamate excitotoxicity and decrease of trophic factors, have broadened potential therapeutic approaches for DR focusing on neuroprotection [15].

#### Reactive gliosis in DR

The astrocytes and Müller cells of the neurovascular unit become activated by neuronal stress and cell death in early DR, which is defined as reactive gliosis [15, 43]. These activated glial cells fail to perform their physiological functions to maintain tissue homeostasis, especially regulation of retinal blood flow, water balance in the neural parenchyma, and maintenance of barrier integrity [44]. In normal conditions, only retinal astrocytes express glial fibrillary acidic protein (GFAP); however, in DR, there is upregulation of this intermediate filament protein in Müller cells, which can be used as a marker of reactive gliosis in the retina [95]. Moreover, activated Müller cells secrete various inflammatory cytokines resulting from the overexpression of innate immune-related pathways [96, 97]. Müller cell studies using diabetic animal models have proposed a potential important role of these cells in retinal water imbalance and vascular abnormalities [58, 98]. In diabetic rats, hyperglycemia induced disruption of Kir4.1 channels in Müller cell end-feet at the interface of retinal capillaries, contributing to the water dysregulation and BRB dysfunction [98]. Another study using a conditional knockout mouse model with disrupted VEGF in Müller cells demonstrated vascular leakage and a significant reduction in the expression of inflammatory biomarkers (tumor necrosis factor alpha (TNFα), intercellular adhesion molecule 1 (ICAM-1) and nuclear factor kappa-B (NF-κB)) as well as depletion of tight junction proteins [58]. This finding has indicated that activated Müller cells may be closely related to retinal inflammation and disruption of the BRB. Likewise, DR also causes Müller cell death, primarily in the form of pyroptosis, which subsequently impairs structural and functional integrity of the neurovascular unit [45].

#### Microvascular pathology in DR

Early in diabetes, patients have demonstrated diminished vasodilation in the retina after flicker light excitation, suggesting the disrupted interactions between local blood flow and neural retina due to hyperglycemia [46, 47]. Although the reduced response indicates the subsequent retinal damage, the patients at this stage are not diagnosed as having DR until the clinical presence of microvascular lesions such as microaneurysms are detected by ophthalmic microscopy [46]. In terms of histopathological alterations at the early stage of DR, vascular basement membrane thickening as well as the demise of pericytes and endothelial cells have been observed [48, 99].

The vascular basement membrane is one of the major components in the neurovascular unit and is crucial to maintain integrity of inner BRB and normal cell-cell or cell-matrix interactions [30]. The thickening of retinal vascular basement membrane, an early microvascular change in diabetes, has been demonstrated to be involved in the development of hyperpermeability and angiogenesis in DR [49, 50]. Furthermore, intravitreal injection of antisense oligos in diabetic rats resulted in the prevention of basement membrane thickening, significant reduction of pericytes loss, acellular capillaries and vascular leakage [100, 101]. It is the imbalance between synthesis and degradation of ECM that contributes to vascular basement membrane thickening. The expression of ECM proteins in the basement membrane are significantly increased, while the degradation of these components by the enzymes are decreased [50]. Due to the alterations of protein components in the thickened basement membrane, cell-cell communication (mainly the communication between vascular endothelial cells and other cells in the neurovascular unit) is impaired, which will subsequently affect the physiological structure and functions of the neurovascular unit [5]. Moreover, the thickened basement membrane damages the inner BRB as a selective barrier, thus leading to vascular leakage [100].

**Fig. 2:**
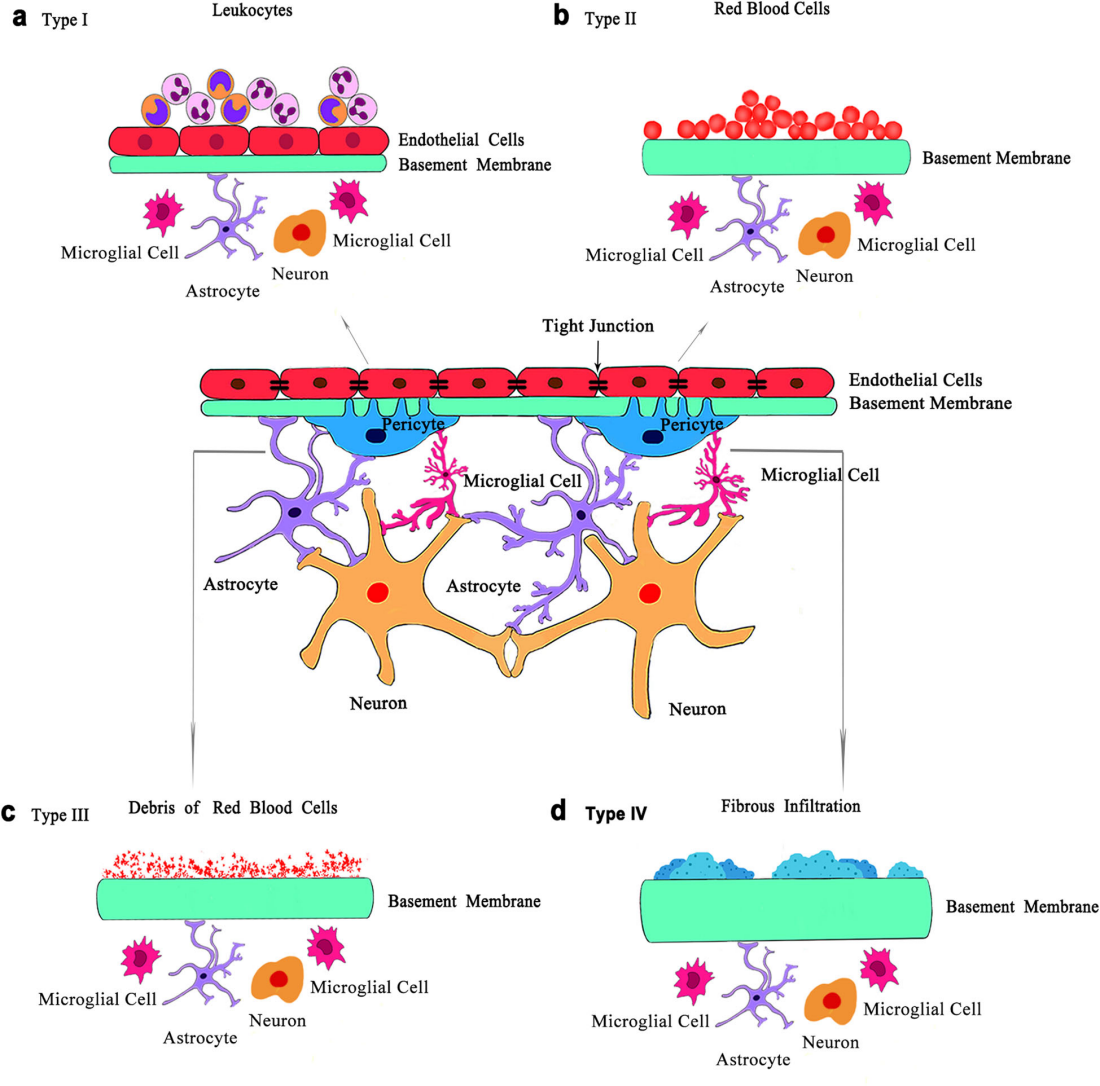
The structure of microaneurysms in DR. Ultrastructurally, there are 4 types of microaneurysms: (**a**) Type I microaneurysms exhibit intact endothelium and basement membrane without the encirclement of pericytes, and circulating leukocytes is extensively accumulated in the lumen; (**b**) Type II microaneurysms are characterized by the absence of both endothelial cells and pericytes, thickened basement membrane, as well as a multitude of red blood cells inside the lumen; (**c**) Type III microaneurysms share similar appearances with Type II microaneurysms, but debris of red blood cells are contained; (**d**) Type IV microaneurysms are in sclerosed forms with heavily thickened basement membrane and fibrous infiltration into the lumen. Moreover, early in DR, retinal neurons undergo apoptotic cell death, accompanied by a decreased number of astrocytes and activation of microglial cells with amoeboid morphology

Loss of pericytes and endothelial cells is also a hallmark of early DR [16]. However, it has been revealed that pericyte loss occurs prior to endothelial cell death in animal models of DR [51]. Pericyte apoptosis and migration away from the original blood vessels may contribute to the diabetes-induced pericyte dropout [13, 51, 52]. A recent research has revealed that in hyperglycemic conditions, pericytes detached from the underlying endothelial cells, migrated off the vessels and formed bridges across two or more capillaries [102]. Increased pericyte bridges were observed in diabetic animal models several months before loss of any pericytes or endothelial cells, suggesting that this is associated with the subsequent pericyte loss and can be used as an indicator of vascular damage [102]. Without the structural support from pericytes, retinal capillaries become weakened, resulting in the formation of microaneurysms and vascular leakage due to the increase of inner BRB permeability [53, 55]. These changes are recognized as the clinical signs in non-proliferative DR patients, of which microaneurysms are one of the first lesions seen in ophthalmoscopic examination and fluorescein angiography [53]. Microaneurysms, outpouchings of the capillary wall, are predominately located on the arteriolar side of the circulation upstream of acellular capillaries [25]. There are several distinct structures of microaneurysms, including intact endothelium without pericytes, loss of both pericytes and endothelial cells, and sclerosed walls of thickened basement membrane (Fig. 2) [54]. The exact mechanism of microaneurysms formation still remains unclear. It is now regarded that the loss of pericytes together with high hydrostatic pressure in capillaries contributes to the formation of microaneurysms [25, 103–105]. The high capillary pressure is caused by the compromised autoregulation in retinal vessels resulting from the death of vascular smooth cells in the arteries and pre-capillary arterioles [25, 54]. Regarding the loss of pericytes, Cai and coworkers demonstrated that angiopoietins (Ang) 2 increased apoptosis of bovine retinal pericytes treated with high glucose via functional Tie-2 receptors, indicating that the Ang-2/Tie-2 system plays an important role in regulating pericytes dropout [106]. Consistent results have been obtained in the animal study, where Ang-2 deficient mice showed reduced pericytes loss by inhibiting hyperglycemia-induced migration of pericytes [51]. Injection of Ang-2 in STZ-induced diabetic mice resulted in the transient elevation of pericyte bridge density, which could be adopted as a predictor of pericyte loss [102]. In mice with selective inactivation of platelet-derived growth factor (PDGF)-B gene in endothelial cells, reduced number of pericytes was observed and proliferative retinopathy developed when more than half of normal pericytes were lost [107]. Moreover, other signaling pathways, such as transforming growth factor-beta (TGFβ) signaling, poly (ADP-ribose) polymerase (PARP)/nuclear factor-kappa B (NF-κB) pathway, polyol pathway etc., may also contribute to diabetes-induced pericytes loss [105]. Although a series of pathways have been revealed to be associated with diabetic pericyte loss, the underlying mechanisms still need to be fully elucidated.

Endothelial cell damage secondary to pericyte dropout is the key pathological feature in DR. Under hyperglycemic conditions, tight junctions between endothelial cells are compromised, which causes the breakdown of the inner BRB, increase of vascular leakage and eventually DME [56, 57]. Reduced expression of tight junction proteins, such as Occludin, claudin-5, ZO-1 and cadherin, has been observed in both human retinal endothelial cells treated with high glucose concentration as well as in diabetic rats [55]. Oxidative stress, cytokines and vascular growth factors alter the expression and organization of tight junction proteins [56]. When the endothelial cells die, capillaries appear as the nude basement membrane tube. Degeneration of these blood vessels is known as retinal vasodegeneration or vasoregression, which leads to the non-perfusion of retinal capillaries and triggers progressive retinal ischemia and hypoxia in DR [13]. By exposure to persistent high levels of blood glucose, endothelial cells undergo apoptosis due to oxidative stress and ischemia [52]. Furthermore, inflammatory mediators released by neighboring neurons, neuroglial and microglial cells may also contribute to endothelial cell death [20]. In response to non-perfusion in the retina, a broad range of angiogenic factors and cytokines, most notably VEGF, are secreted by ischemic retinal neurons to facilitate the formation of abnormal new blood vessels [8]. This so-called neovascularization is the central microvascular characteristic of proliferative DR and causes irreversible blindness resulting from extensive bleeding in the vitreous of these fragile neovessels. Vasoregression and neovascularization are regulated by a highly complicated system in which several crosslinked signaling pathway (such as Wnt, Notch and NF-κB) and imbalance between angiogenic and anti-angiogenic factors are involved [108, 109].

#### Immuno-inflammation in DR

Local inflammation acts as a pivotal risk factor in the development and progression of DR, and thus DR has also been regarded as chronic inflammatory disorder [15, 59, 62, 65, 66]. With progression of the disease, inflammatory reactions including activation of immune cells and production of diverse inflammatory cytokines are present within the retina [15]. At the early stage of DR, the adherence of circulating leukocytes such as neutrophils and monocytes to vascular walls is enhanced by the increased expression of ICAM-1 and P-selectin in endothelial cells [60]. This enhanced leukocyte-endothelial interactions, which is known as leukostasis, can induce injury to retinal vascular endothelium and neighboring tissue through capillary occlusion and non-perfusion as well as secretion of inflammatory molecules [61]. The genetically modified mice that lack expression of ICAM-1 and CD18 have exhibited fewer adherent leukocytes after streptozotocin treatment, leading to protection against damage of endothelial cells and hyperpermeability of blood vessels [59].

Apart from intravascular immune cells, microglial cells, the resident immune cells in the retina, are activated by advanced glycation end products and reactive oxygen species (ROS) in DR [62–64]. The activation can be observed by the morphological changes, from ramified to amoeboid morphology. At the early stage of DR, the major effects of activated microglia are phagocytosis of apoptotic cells, clearance of debris and secretion of neurotrophic molecules [65]. Nevertheless, as DR progresses, persistently activated microglial cells become detrimental to retinal neurons and vasculature via release of inflammatory cytokines [66]. TNFα and interleukin-1beta (IL-1β) could cause retinal neuron death and vascular endothelial impairment with subsequent breakdown of the inner BRB through caspase-3 activation [65, 67]. Vincent and Mohr also demonstrated that IL-1β could lead to the apoptosis of retinal capillary cells via activation of caspase-1. Furthermore, mice deficient in IL-1β receptor suppressed the caspase activation and capillary degeneration in the diabetic retina of mice [110]. IL-8 and monocyte chemoattractant protein 1 (MCP-1) are chemotactic factors that recruit neutrophil and monocytes to the inflammatory sites in the retina, respectively, increasing their infiltration within the retina [68, 69]. Moreover, IL-6 induces production of VEGF by activating the STAT3 pathway in vascular endothelium, promoting vascular leakage and DME [69]. These events act in concert to create an inflammatory environment that poses pathological changes in DR.

#### Dysfunction of RPE and choroid in DR

Although more attention has been paid to the diabetes-induced deterioration of retinal vasculature and neurons, DR also impacts RPE and its underlying choroid. RPE cells form a continuous and polarized cell monolayer with a variety of physiological functions, including transportation of nutrients, ions and water between the photoreceptors and choriocapillaris, phagocytosis of photoreceptor outer segments, conversion of all-trans-retinal into 11-cis-retinal in the visual cycle, absorption of light and protection against photooxidation, and secretion of a series of essential factors to maintain retinal integrity [111]. In diabetic rats, activity of the Na^+^-K^+^-ATPase, the major energy source for active transportation of ions and water in RPE cells, was significantly decreased [70]. Therefore, the transport of water driven by a transport of K^+^ and Cl^-^ from the subretinal space to the blood was damaged, favoring the macular edema in DR [34, 71]. The key proteins involved in the visual cycle, such as IRBP and RPE65, have been reported to be significantly reduced in both diabetic animal models and patients, indicating the decrease of 11-cis-retinal supplied to the photoreceptors [71, 72]. Additionally, various factors secreted by RPE cells, such as VEGF, PDGF, TNFα, IL-6, IL-8 etc., are upregulated, contributing to the development and progression of DR [73]. Most importantly, under hyperglycemic conditions, the integrity of outer BRB formed by RPE cells and their intercellular tight junctions is disrupted with reduced expression of tight junction proteins (claudin-1 and occludin), leading to fluid leakage from the choriocapillaris [34, 74, 75]. In hyperglycemic rats, the breakdown of the outer BRB has also been confirmed with wider tight junctions and large holes observed between cells when examining another tight junction protein-zona occludens (ZO-1) [34, 76]. Severe macular detachment resulting from RPE barrier dysfunction has been observed in a third of DME cases, suggesting the co-occurrence of inner and outer BRB breakdown in the progression of DME [55, 112]. It has been reported that the outer BRB accounts for more than one third of total vascular leakage in diabetic retinas [71]. Meanwhile, impaired fluid clearance from the neural retina due to RPE dysfunction occurs, which, along with outer BRB destruction, plays an important part in DME [113]. Diabetic rats have also presented increased accumulation of immune cells in the outer retina and apoptosis of photoreceptors caused by damaged RPE [114]. Diabetic choroidal degeneration has been reported in both patients and animal models, and exhibits decreased choroidal thickness, increased Bruch’s membrane thickness, aneurysms and choroidal neovascularization [77–79]. The further understanding of RPE and choroidal pathology in DR still needs clinical and experimental investigation.

### Potential therapeutic strategies for DR

Although therapies for DR have been greatly improved for the past decades, there is still a large demand in additional therapeutic options. Current treatments focus exclusively on the advanced stage of DR, in which irreversible injuries in the retina have occurred [3, 5]. Therefore, therapies that target early pathology or have preventive actions are highly required. Here, some of the novel therapeutic approaches based on pathology of the neurovascular unit in DR will be briefly discussed.

#### Neurotrophic factors

Neurotrophic factors are beneficial for neuronal growth, differentiation and neurovascular interactions [115, 116]. Reduction of these molecules has been considered to play a crucial role in retinal neurodegeneration [115–117]. A series of neurotrophic factors, such as PEDF, brain-derived neurotrophic factor (BDNF), insulin and insulin-like growth factor-1 (IGF-1), SST as well as glucagon-like peptide-1 (GLP-1), is of great importance to provide neuroprotection in DR [118–124].

PEDF secreted by RPE cells, vascular endothelial cells, glial cells, Müller cells, and neurons shows both neuroprotective and anti-angiogenic effects under hyperglycemic conditions through inhibition of oxidative stress, inflammation as well as glutamate excitotoxicity [120, 125, 126]. In both patients with proliferative DR and diabetic rats, PEDF levels were decreased in the retina [120, 125]. Furthermore, early in the course of experimental DR, intravenous administration of PEDF could ameliorate retinal neuron damage and reduce increased expression of VEGF via antioxidant effects [120]. In addition, topical administration of PEDF has demonstrated reduced retinal ganglion cell death, Müller cell activation and microvascular leakage in a mouse model of DR [127]. It has been further confirmed *in vitro* that PEDF secreted by Müller cells promoted survival of retinal ganglion cells via activation of the STAT 3 pathway [128, 129].

BDNF is crucial as it supports tje survival of amacrine cells and retinal ganglion cells. Similar to PEDF, reduction of BDNF levels in the retina, which was associated with the degeneration of dopaminergic amacrine cells, was observed in diabetic rats induced by streptozotocin [130]. Intravitreal injection of BDNF have promoted density of dopaminergic amacrine cells, indicating its protective role in retinal neurodegeneration [130, 131]. Delivery of BDNF with recombinant adeno-associated virus has been beneficial in that it improved retinal ganglion cell survival and function in an experimental animal model of DR [121, 131]. However, the effects of BDNF in the retina have been reported to be dependent on various concentrations. At optimal concentrations, BDNF may exhibit a neuroprotective role in DR, while higher concentrations might elicit inflammatory responses [131, 132].

Insulin and its receptor signaling pathway provide support to maintain the normal state of retinal neurons. Impairment of insulin and its receptor signaling pathway contributes to the neuroretinal deterioration at the early stage of DR [122, 133]. Systemic and local (intravitreal and subconjunctival) administration of insulin has been proven to regain the insulin receptor activity, which in turn prevent the loss of retinal neurons [122, 134]. Therefore, biodegradable hydrogels or chitosan nanoparticles that can be implanted subconjunctivally has been developed to supply a sustained release of insulin to the rat retina with no negative effects [135, 136]. IGFs are the hormones involved in the progression of diabetes-induced retinal disease. In particular, decreased IGF-1 mRNA has been observed in the initial stage of both clinical and experimental diabetic eyes [137]. Diabetic rats treated with an IGF-1 analog have shown prevention of early retinal biochemical abnormalities related to DR pathogenesis [138].

SST is produced in the retina and exhibits neuroprotective and anti-angiogenetic properties in conjugation with its receptors, especially with the SST receptor 2 [123]. Low SST level, an early event of DR, has been reported to stimulate apoptosis of neurons and glial activation in the retina through extracellular accumulation of glutamate and downregulation of glutamate transporter [82, 123]. Therefore, SST has been applied to diabetic murine models via topical administration and found to ameliorate retinal neurodegeneration and gliosis by reducing glutamate excitotoxicity and inducible nitric oxide synthase (iNOS) level [123, 139]. Furthermore, a multi-center, randomized controlled clinical trial (EUROCONDOR) has shown that topical administration of SST could stop worsening of pre-existing neuroretinal dysfunction, although no retinal neuroprotection was observed in subjects included in this clinical trial [140].

GLP-1 is secreted by L cells (specific endocrine cells) in the small intestine in response to food intake. Apart from its anti-hyperglycemic effects, GLP-1 also exhibits neuroprotective functions in the central and peripheral nervous system [141]. Moreover, both GLP-1 and its receptor GLP-1R have been detected in human and murine retinas, indicating its potential neuroprotective role in the retina [142]. Expression of GLP-1 was reduced in the retina of diabetic patients when compared with controls, and similar findings were observed in diabetic animal models as well [142–145]. In animal models of DR, administration of GLP-1, GLP-1R agonists, or dipeptidyl peptidase IV (DDP-IV) prevented apoptosis of retinal neurons, loss of pericytes, and decrease of retinal thickness, resulting in the improvement of retinal functions as evaluated by ERG [124, 143, 146–148]. The underlying mechanisms might be inhibition of oxidative stress and decrease of glutamate excitotoxicity [143, 147, 148]. However, in clinical trials, GLP-1R agonists did not show protective effects on DR. On the contrary, a nonsignificant increase in the progression of DR was reported in the LEADER trial (liraglutide), and significant increase of DR incidence was shown in the SUSTAIN-6 trial (semaglutide) [149, 150]. Simo and Hernandez explained the possible reasons of the discrepancies, including short follow-up duration, lack of grading of DR, and rapid lowering of HbA1c [141].

#### Antioxidants

Oxidative stress has been shown to play a central role in the development of DR in many studies [15, 52, 56, 63, 120, 151, 152]. Thus, usage of antioxidants may be adopted as potential treatment options for DR. Flavonoids and carotenoids are examples of phytochemicals that are able to counteract diabetes-induced oxidative stress in the retina.

Flavonoids have antioxidant, anti-inflammation and anti-angiogenic functions, and effects of selected flavonoids have been examined for the prevention and/or treatment of DR [153–158]. For instance, quercetin could prevent oxidative stress, neuroinflammation and neuronal apoptosis in the retina of diabetic rats [153, 154]. After administration of quercetin, the rats have exhibited restored antioxidant activities, decreased expression of inflammatory cytokines and reactive gliosis as well as reduction of retinal ganglion cell apoptosis [153, 154]. In addition, treatment of quercetin has inhibited overexpression of aquaporin-4 (protein forming water-specific channel in the membrane), which has been thought to contribute to neuroglial edema [153]. A number of studies have been carried out to investigate the antioxidant and anti-angiogenic role of curcumin in the treatment of diabetic complications, including DR. Administration of curcumin in diabetic rats led to the reduction of VEGF expression; inhibition of oxidative stress; protection of inner nuclear layer cells, retinal ganglion cells and Müller cells; as well as prevention of glutamine synthetase downregulation [155–157]. In addition, treatment with curcumin has also diminished the levels of inflammatory mediators, such as TNFα and VEGF, as well as limiting structural degeneration and increased thickness of capillary basement membrane in experimental diabetic retina [158].

Lutein and zeaxanthin, the carotenoids highly concentrated in the retina, play a major role in filtering blue light, preventing oxidative damage and protecting neural retina [159–164]. In non-proliferative DR patients, the serum concentration of lutein and zeaxanthin was significantly lower than individuals without diabetes [160]. A three-month supplementation of lutein (6mg/day) and zeaxanthin (0.5mg/day) resulted in a significant increase of blood lutein and zeaxanthin level, as well as the improvement in visual acuity, contrast sensitivity and DME in non-proliferative DR [160]. These data were consistent with the conclusions drawn from the administration of 10mg/day lutein for 36 weeks in patients with non-proliferative DR [161]. In type 2 diabetics, an increased thickness in the central fovea and improved retinal response density were observed after a two-year supplementation of combined lutein (10mg/day), zeaxanthin (2mg/day) and meso-zeaxanthin (10mg/day) [162]. The studies using diabetic animal models to investigate lutein and zeaxanthin effects have also demonstrated protection from diabetes-induced retinal apoptosis, abnormal capillaries formation, and visual dysfunction through decreased ROS, and inflammatory factors such as VEGF, IL-1β, and NF-ΚB in the retina [159, 163, 164].

#### Anti-inflammation agents

As mentioned above, elevation of inflammatory cytokines, such as TNFα, IL-1β, IL-6, IL-8 and VEGF, are associated with DR pathogenesis [58, 65, 67–69, 110]. Moreover, upregulation of ICAM-1 leads to leukostasis in retinal vessels, which may subsequently contribute to neuroretinal ischemia, favoring neurodegeneration in the retina [58–60]. Agents that counteract the impact of inflammatory molecules, therefore, may be considered an option for DR therapy.

TNFα antagonist, etanercept, has shown beneficial effects in suppressing retinal cell apoptosis, increase of ICAM-1 expression and loss of vascular endothelial cells and pericytes in experimental diabetic animal models [165–167]. Nevertheless, after three months of intravitreal injection of etanercept, no significant improvement was observed in patients with refractory DME [168]. Likewise, a pilot clinical study using intravitreal administration of adalimumab and infliximab (the other two TNFα inhibitors) has not revealed any benefits for the eyes with refractory DME. Furthermore, 2mg infliximab administration could stimulate serious intraocular inflammation [169]. Hence, clinical trials failed to show beneficial effects of TNFα inhibitors, albeit TNF’s role in disrupting the neurovascular unit.

Suppressor of cytokine signaling (SOCS) 1 belongs to the family of SOCS proteins that regulate cytokine responses through a negative feedback loop [170]. SOCS 1 plays a crucial role in negatively regulating TNFα and IL-6, which are closely related with DR. Two weeks after topical administration of SOCS 1-derived peptide in db/db mice, reactive gliosis and presence of activated microglial cells were significantly decreased, indicating reduced glial activation [171]. In terms of proinflammatory cytokines, retinal levels of IL-1β, IL-6 and perivascular TNFα deposition were dramatically reduced in the SOCS 1-derived peptide treated group in comparison with the vehicle group. In addition, SOCS 1-derived peptide ameliorated abnormal retinal functions induced by diabetes due to preservation of the neural retina [171].

Minocycline, one of tetracycline derivatives, has also been studied for the treatment of DR due to its inhibition of microglial activation and PARP-1 expression properties [67, 172–174]. Minocycline could reduce inflammatory molecules including TNFα and IL-1β released by activated microglial cells, suggesting its potential role in preventing neuroretinal and microvascular atrophy in DR [172]. Data from the *in vitro* study was further confirmed by the findings in the streptozotocin-induced diabetic rat model with prevention of retinal neuron death triggered by activated microglia through decreased caspase-3 activation and abnormal histone methylation levels [67, 173]. Diabetic rats fed with minocycline daily for 8 weeks exhibited decreased apoptosis of retinal cells through inhibition of PARP-1, which was abnormally activated by diabetes-induced DNA damages [174]. Furthermore, a single-centered, phase I/II clinical trial has been conducted to evaluate the effects of oral administration of minocycline on DME. Catherine and colleagues treated the patients with 100mg minocycline twice a day for up to six months. After the treatment, improvement of central macular edema, vascular leakage and visual function was observed in all five participants [175]. Doxycycline, another tetracycline derivative, has been tested on 33 patients with mild to moderate non-proliferative DR for two years. However, no significant protective effects were detected in diabetes-induced visual function and retinal injury [176].

#### Cell-based therapeutic strategies

Since the loss of several different cells in the retina is involved in the pathology of DR, cell replacement using stem cells has drawn attention from researchers in the past years. Mesenchymal stem cells (MSC), hematopoietic stem cells (HSC), endothelial progenitor cells (EPC), and induced pluripotent stem cells (iPSC) have been evaluated in either preclinical or clinical studies for the treatment of DR [177].

MSC are multipotent stem cells and can be easily harvested from the bone marrow, adipose tissue, dental pulp, umbilical cord blood and Wharton’s jelly, making them a promising cell source for cell therapy of DR [99]. In a rat model with pathological features of early DR, intravitreal injection of MSC significantly restored the retinal function by improving b-wave amplitudes in ERG and protected retinal structure by decreasing vascular leakage and apoptosis of cells around the vessels [178]. Treatment of diabetic mice with MSC completely attenuated the death of retinal ganglion cells which is an early event in the onset of DR, indicating the therapeutic potential of MSC at the initial stage of DR [179, 180]. Furthermore, MSC administration suppressed diabetes-induced damage to the neurovascular unit of the retina by reducing ROS levels and increasing neurotrophic factors including nerve growth factor, basic fibroblast growth factor and glial cell line-derived neurotrophic factor [179]. Another study performed by Yang and colleagues has shown differentiation of MSC into photoreceptors and glial-like cells in the retina, resulting in improved integrity of the BRB in diabetic rats [181]. In one ongoing clinical trial (NCT01736059), the safety and feasibility of intravitreal injection of bone marrow MSC is being investigated in individuals suffering from irreversible visual loss caused by retinal degenerative diseases or retinal vascular diseases, including DR. The safety and efficacy of intravenous administration of autologous bone marrow MSC has been evaluated by Gu et al., demonstrating decrease of macular thickness and significant improvement of best corrected visual acuity in non-proliferative DR patients, but not in proliferative DR patients [182].

EPC are regarded as a heterogeneous group of cells which can differentiate into endothelial cells directly or induce angiogenesis via paracrine action to restore impaired vasculature [183]. The functions of human EPC in ischemic retinal diseases have been evaluated in animal models of DR, ischemia/reperfusion and oxygen-induced retinopathy [184–186]. Transplantation of IL-10-transfected EPC from the rat bone marrow led to significant improvement of retinal structure and reduced retinal vascular permeability through inhibiting inflammation in diabetic rats [184]. Intravitreal and intravenous administration of healthy human EPC from bone marrow or peripheral circulation could rapidly settle down and integrate into damaged retinal vasculature, suggesting the use of these cells as a potential therapy for DR [185, 186]. However, EPC from diabetic patients failed to repair vascular impairment in the retina of murine models as mentioned above [185]. Therefore, a variety of approaches have been adopted to restore functions of EPC in patients with diabetes, including inhibition of TGFβ1, treatment with peroxisome proliferator-activated receptor γ and δ, improvement of EPC mobilization with stromal cell-derived factor-1 [183].

Dysfunction of HSC resulting from the disturbance of the vasoprotective axis of the renin angiotensin system is a feature of DR, thus, making the activation of protective renin angiotensin system within HSC a treatment option for DR [187]. Human iPSC derived from cord blood could generate vascular progenitor cells, which exhibited homing and integration into injured retinal vessels for up to 45 days [188]. Human iPSC-derived endothelial cells formed more complex vascular networks *in vitro* and integrated into host regenerating retinal vessels in a mouse model of ischemic retinopathies when compared to human mature endothelial cells, suggesting its superior angiogenic potential in ischemic retinopathies (such as proliferative DR) [189]. One clinical trial (NCT03403699) is evaluating the ability of human iPSC to generate endothelial cells and pericytes for revascularization of vasodegenerative capillaries occurring in DR.

#### Other treatment approaches for improving retinal neurovascular coupling

Activity of neurons in the inner retina is closely coupled with the retinal blood flow in physiological conditions [8, 9, 11, 190]. Indeed, it is the interaction between neural retina and microvascular networks that supply more oxygen and nutrients to match the increased neuronal activity in the retina [9, 11]. For instance, visual stimulation such as flicker light stimulation could induce increased metabolism of retinal neurons, which in turn dilates retinal blood vessels, especially the smaller vessels via neurovascular coupling, thus eventually leading to the increase of retinal blood flow [191, 192]. However, this response, known as functional hyperemia, is diminished in early DR due to the impairment of retinal neurovascular coupling [16, 193].

Early in DR, the vascular response in neurovascular coupling is compromised, depriving the retinal neurons of adequate oxygen and nutrients, and thus contribute to the death of neuronal cells. Increased iNOS produces high nitric oxide (NO) levels in the diabetic retina, which would cause defective interconnections between neuronal activity and arteriole dilation via inhibition of glia-derived dilatory molecules [16]. Moreover, up-regulation of NO concentrations has shown an association with increased number of acellular capillaries and pericytes loss in streptozotocin-induced diabetic mice; while all the abnormalities have been prevented in diabetic iNOS knockout mice [194–196]. In patients with DR, high levels of NO generated by iNOS led to neurotoxicity and angiogenesis in the retina [194, 197]. Hence, suppression of iNOS and reduction of NO levels may be useful in promoting neurovascular coupling and ameliorate damages to neurons, pericytes as well as endothelial cells. Administration of the iNOS inhibitor, aminoguanidine, recovered vessel dilations in experimental DR [198]. Sesamin, a natural compound, could restrain the progression of diabetic retinal damage through the reduction of hyperglycemia and inflammation in the retina of diabetic mice including inhibition of iNOS [199].

Dopamine is a pivotal neurotransmitter in the retina and brain. Reduced synthesis of dopamine has been proved to be closely associated with diabetes-induced malfunctions of retinal neurons in both diabetic animal models and patients [200–202]. Treatment with L-3,4-dihydroxyphenylalanine (L-DOPA), a precursor of dopamine, has partially restored visual functions, showing preservation of delayed b-wave latencies and oscillatory potential as well as reduced contrast sensitivity thresholds and spatial frequency in STZ diabetic mice [200, 201]. In diabetics without clinically visible retinal vascular defects, oscillatory potential implicit time was delayed, which was preserved after a two-week treatment of Sinemet (L-DOPA plus carbidopa), suggesting that L-DOPA may be one potential candidate for treating early DR [202].

Endothelin-1(ET-1), mainly synthesized in the vascular endothelium, is one of the most potent vasoconstrictor peptides. It has been reported that elevated serum concentration of ET-1 is intimately correlated with microangiopathy in Type 2 diabetic patients [203]. Moreover, through ET_A_ receptors, ET-1 makes contribution to retinal hemodynamic disruption and pathogenesis of DR in experimental animal models, indicating its evident role in the interaction between vascular abnormalities and neural retina damage [203]. In this regard, blocking ET-1 actions through ET_A_ receptors may be considered a potential treatment for DR. Indeed, atrasentan (the ET_A_ receptor antagonist) attenuated the reduction of red blood cell velocity, flow rate and wall shear rate, loss of pericytes, percentage of acellular capillaries as well as protected the inner retina in several animal models of diabetes [204, 205]. Recent studies have also shown retinal neurodegeneration via the activation of ET_B_ receptors, the other type of ET-1 receptor, in several animal models [206]. Therefore, inhibition of ET_B_ receptor activation could prevent degeneration of the neural retina. A study conducted by Patricia and colleagues has reported that bosentan (a dual ET-1 receptor antagonist) significantly decreased glial activation and apoptotic cells in all retinal layers of db/db mice, making it an ideal candidate for treating DR at an early stage [206].

In addition to the aforementioned therapeutic approaches (Table 2), other treatments including implantation of whole retinal cell sheets, use of bioelectronic prostheses transplanted in the subretina, modification of other types of retinal cells to generate retinal neurons, use of agents to block glutamate signaling pathway and so on are also under investigation [207–210].

**Table 2:** Summary of potential treatments for diabetic retinopathy (DR)

Types of Treatments		Major Mechanisms	References
Neurotrophic factors	PEDF	• Neuroprotection • Anti-oxidative stress • Anti-inflammation • Anti-angiogenesis • Reduction of blood glucose, glutamate excitotoxicity, iNOS level	[120, 125, 129]
	BDNF		[121, 130, 131]
	Insulin & IGF-1		[122, 134, 138]
	SST		[123, 139]
	GLP-1		[124, 143, 146–148]
Anti-oxidants	Flavonoids (Quercetin, Curcumin)	• Anti-oxidative stress • Anti-inflammation • Anti-angiogenesis • Decrease of retinal neurons apoptosis	[153–158]
	Carotenoids (Lutein, Zeaxanthin)		[160–164]
Anti- inflammation agents	TNFα antagonist (Etanercept, Adalimumab, Infliximab)	• Decrease of retinal neurons, pericytes and endothelial cells loss • Reduction of inflammatory cytokines level • Inhibition of glial activation and PARP-1	[165–167]
	SOCS 1		[170, 171]
	Tetracycline derivatives (Minocycline, Doxycycline)		[172–175]
Cell replacement	MSC	• Anti-oxidative stress • Anti-inflammation • Decrease of retinal neurons, pericytes and endothelial cells loss • Increase of neurotrophic factors • Differentiation into retinal and vascular cells	[178–181]
	EPC		[184–186]
	HSC		[187]
	iPSC		[188, 189]
Other treatments	Inhibition of iNOS (iNOS knockout Aminoguanidine, Sesamin)	• Decrease of retinal neurons, pericytes and endothelial cells loss • Anti-inflammation • Reduction of blood glucose	[195–199]
	L-DOPA	• Preservation of retinal functions	[200–202]
	ET-1 receptor antagonist (Atrasentan, bosentan )	• Decrease of retinal neurons, pericytes and endothelial cells loss • Inhibition of glial activation • Neuroprotection	[204–206]

*PEDF*= pigment epithelium-derived factor; *BDNF*= brain-derived neurotrophic factor;* IGF-1*= insulin-like growth factor-1; *SST*= somatostatin; *GLP-1*= glucagon-like peptide-1; *Inos*= inducible nitric oxide synthase; *TNFα*= tumor necrosis factor alpha; *SOCS*= suppressor of cytokine signaling; *PARP-1*= poly (ADP-ribose) polymerase-1; *MSC*= mesenchymal stem cells; *EPC*= endothelial progenitor cells; *HSC*= hematopoietic stem cells; *iPSC*= induced pluripotent stem cells; *L-DOPA*= L-3,4-dihydroxyphenylalanine;* ET-1*= endothelin-1

There is still one issue that needs to be considered: the appropriate administration route for treating early DR. It would be too much aggressive to choose intravitreal administration at the early stage of DR, although this route provides direct, local and relatively prolonged pharmacological effects on the retina [211]. In addition, certain complications have been observed, including cataract, endophthalmitis, retinal detachment, vitreous hemorrhage, etc [212]. The therapeutic effects of systemically administered drugs targeting the posterior eye are limited by the BRB as evidenced by the limited penetration of drugs (only 1-2%) across the BRB and into the intraocular tissue [212]. It seems that drug delivery through the topical route might be the suitable administration route in the initial stage of DR because of its convenience, non-invasiveness and high patient compliance. However, the concentration of drugs that reaches the retina is very low (less than 5% of the administered dosage), owing to the blood-ocular barriers, nasolacrimal drainage and tear dilution [211–214]. Recently, Simό and coworkers have demonstrated that a number of drugs administered topically could reach the retina at effective concentrations in animal models of DR [123, 124, 143, 206, 215]. But, to best of our knowledge, there is no supporting data from clinical trials. Now, scientists from multidisciplinary teams are trying to develop drug delivery systems that target the posterior segment of the eye via the topical route. One of them is polymeric nanoparticles, namely Mucus Penetrating Particles, which are capable of bypassing all ocular barriers and delivering drugs to the retina due to the enhanced migration through mucus [216, 217]. Another technology owned by Macregen Inc. is to add cell-penetrating peptides in the eye drop, which can penetrate across the corneal barriers and reach the posterior of the eye, achieving the equivalent therapeutic efficacy as intravitreal injection [218, 219]. Additionally, a hydrogel ring made of hydroxyethyl methacrylate has been designed to deliver topically administered drugs to the retina/choroid via the sclera and conjunctiva [219, 220]. With the development in drug delivery systems, topical administration would be the preferred choice to deliver drugs to the posterior eye in the early course of DR.

## Conclusions

DR is one of the most common causes of irreversible vision loss around the world. For a long time, DR has been considered as a microvascular disease featured with vascular leakage and capillary occlusion at the early non-proliferative stage and neovascularization at the late proliferative stage. To date, current treatments for DR mainly focus on retinal microvascular lesions, including intravitreal administration of anti-VEGF as the first-line therapy and laser photocoagulation, intravitreal corticosteroids, and vitreous surgery as adjuvants. However, these interventions are invasive, expensive and visits to the ophthalmologist’s clinic are needed at certain intervals. Therefore, new therapeutic strategies drawing from a deeper understanding of DR pathogenesis are required. A growing body of evidence based on clinical and experimental studies suggests that rather than DR being solely a microvascular disorder, DR can be an intricate disorder with the disruption of vascular cells, neurons, glia, local immune cells, RPE cells as well as interconnections among these cells. This concept has broadened our understanding of the cells involved in the pathophysiology of DR and provided additional clues for developing novel therapies to prevent or halt the disease at the initial stage in comparison with current treatment modalities targeting on DME and proliferative DR at the advanced stage. A variety of novel treatment options focusing on the components of the neurovascular unit and their interactions, such as neurotrophic factors, antioxidants, anti-inflammatory agents, cell replacement and so on, have demonstrated protective effects on retinal functions and structure in both animal models and clinical trials. Although numerous potential interventions have shown exciting results, further studies to evaluate not only the long-term effects and safety of these interventions, but also the appropriate drug delivery routes are still needed before implementation in clinical practice.

## Data Availability

Not applicable

## Abbreviations

BDNF: brain-derived neurotrophic factor
BRB: blood-retinal barrier
DDP-IV: dipeptidyl peptidase IV
DME: diabetic macular edema
DR: diabetic retinopathy
ECM: extracellular matrix
EPC: endothelial progenitor cells
ERG: electroretinogram
ET-1: endothelin-1
GFAP: glial fibrillary acidic protein
GLP-1: glucagon-like peptide-1
HSC: hematopoietic stem cells
ICAM-1: intercellular adhesion molecule 1
IGF-1: insulin-like growth factor-1
IL-1β: interleukin-1beta
INL: inner nuclear layer
iNOS: inducible nitric oxide synthase
IPL: inner plexiform layer
iPSC: induced pluripotent stem cells
IRBP: interphotoreceptor retinol binding protein
L-DOPA: L-3,4-dihydroxyphenylalanine
MCP-1: monocyte chemoattractant protein 1
MSC: mesenchymal stem cells
NF-κB: nuclear factor kappa-B
NFL: nerve fiber layer
NO: nitric oxide
PARP: poly (ADP-ribose) polymerase
PDGF: platelet-derived growth factor
PEDF: pigment epithelium-derived factor
ROS: reactive oxygen species
RPE: retinal pigment epithelium
SOCS: suppressor of cytokine signaling
SST: somatostatin
TGFβ: transforming growth factor-beta
TNFα: tumor necrosis factor alpha
VEGF: vascular endothelial growth factor
ZO-1: zona occludens 1
